# On Revelations and Revolutions: Drinking Ayahuasca Among Palestinians Under Israeli Occupation

**DOI:** 10.3389/fpsyg.2021.718934

**Published:** 2021-08-27

**Authors:** Leor Roseman, Nadeem Karkabi

**Affiliations:** ^1^Centre for Psychedelic Research, Imperial College London, London, United Kingdom; ^2^Department of Anthropology, University of Haifa, Haifa, Israel

**Keywords:** ritual, psychedelics, Badiou’s Being and Event, Israeli-Palestinian conflict, New Age, activism, DMT, prophecy

## Abstract

The ritualistic use of ayahuasca can induce a feeling of unity and harmony among group members. However, such depoliticized feelings can come in the service of a destructive political *status quo* in which Palestinians are marginalized. Through 31 in-depth interviews of Israelis and Palestinians who drink ayahuasca together, and through participatory observations, such rituals were examined. In this setting marginalization was structurally rooted by the group’s inability to recognize Palestinian national identity or admit the ongoing Israeli injustice toward Palestinians. Although the groups avoided politics, they still find their way into these rituals. This happened through occasional ayahuasca-induced revelatory events, in which individuals were confronted with a pressing truth related to the oppressive relations between Jewish Israelis and Palestinians. Three case studies of such revelatory events are described in this paper. Affected by emotions of pain, anger, and guilt, these participants developed resistance toward the hegemonic Israeli ritual structure. This was followed by an urge to deliver an emancipatory message to the rest of the group, usually through a song. Moreover, affected subjects developed a long-lasting fidelity to the *truth* attained at these events. In time, this fidelity led to the expansion of ayahuasca practices to other Palestinians and the politicization of the practice. The article draws on Badiou’s theory in *Being and Event* (1988) to analyze the relations between the Israeli ritual structure, the Palestinian revelatory event, and the emancipatory fidelity that followed. Badiou’s theory elucidates the egalitarian revolutionary potential, which is part of the sociopsychopharmacology of psychedelics.

## Introduction

During an ayahuasca ritual on the Jewish holiday of Yom Kippur (Day of Atonement), Ruqaiya had a painful historical revelation. She saw cycles of war, blood flowing onto the land, and mothers who sacrifice their children.^1^ As a Palestinian citizen of Israel who had denied her national affiliation in the past, she finally recognized the longstanding injustice of the Israeli occupation of her people. Embodying the agony of the land, she expressed her anger in a song, which interfered with the harmony of the ritual. Singing *al-Fatiha* (the opening verses of the Quran) with great vigor, she attempted to deliver an emancipatory message as a Muslim to the Jewish Israeli participants attending the ritual: fasting on Yom Kippur is not enough, since a political reparation is necessary for their atonement. While some of the Jewish Israeli participants rejected her message, the political intensity of the moment was unavoidable. Ruqaiya’s particular feelings that evening about Palestinian subjugation were associated with a universal truth of liberation and equality for all. Her loyalty to this revelatory event changed her life and gave her a new sense of mission. She became a facilitator of ayahuasca rituals that incorporate Palestinian cultural elements and political awareness into the practice.

Ruqaiya’s delivery of her vision was unusual in the context of organized Israeli ayahuasca rituals in which Palestinians participated. Kept strictly “apolitical,” the gatherings, directed by the Israeli organizers, carefully maintained a discourse of denationalized unity. “Mysticism unifies, while politics divide,” was a statement occasionally heard while conducting fieldwork in such mixed groups. However, politics were unavoidable, even in such “protected” settings. While participants often experienced visions related to the political violence in Palestine/Israel, some were so deeply affected by what they felt that they were compelled to speak truth to the group. Based on interviews with individuals who went through such life-changing events, this inquiry was initiated to problematize the discourse about peace in these rituals and examine how psychedelics may provoke the formation of politicized subjectivities.

Ayahuasca is a psychedelic Amazonian brew whose main pharmacologically functional components are two plant-derived ingredients: dimethyltryptamine (DMT), a classic tryptamine psychedelic, and a mix of monoamine oxidase inhibitors (MAOIs) that prevent the breakdown of DMT when consumed orally (Rivier and Lindgren, 1972). The effect of psychedelics is known to be context dependent (Carhart-Harris et al., 2017; Hartogsohn, 2017, 2020), and psychedelics are known to produce a myriad of intense experiences (Dobkin de Rios, 1973; Shanon, 2002); they therefore are referred to as “non-specific amplifiers” (Grof, 1975/2016). The cultural influence on the visionary experience (La Barre, 1938/1975; Wallace, 1959; Langdon, 1979) has recently been termed the “socialization of hallucinations” (Dupuis, 2021). It is suggested that the goals of the practice—for example, the ways in which healing is achieved—can influence the content of the psychedelic experience. For instance, within a Christian Amazonian rehabilitation program, ayahuasca healing of “possessed” patients is mediated through the experience of exorcism (Dupuis, 2018). In comparison, within Western clinical trials, psychedelic healing is mediated by the *mystical union* (MacLean et al., 2012; Roseman et al., 2017). In both cases, elements of the context and the intentions behind the practice increase the possibility that the desired experience will take place.

The mystical union is an experience in which one feels interconnectedness with others and the universe (Pahnke and Richards, 1966; Shanon, 2002; MacLean et al., 2012). A fusion of the self with a larger whole, the mystical union represents peace, harmony, oneness, and acceptance. In the context of peacebuilding, such representations can at times strengthen relations between participants but may also conceal, and even deny, the violence that one group inflicts on another. According to research in intergroup contact, those in power seek harmony, friendship, and “connecting as humans,” while those with less power seek liberation and decolonization (Abu-Nimer et al., 2007; Saguy et al., 2009; Maoz, 2011). Indeed, for Palestinians, who suffer from Israeli settler colonization, occupation, and subjugation, “peace” does not necessarily equal harmony. Rather, in their contact with Israelis, they seek justice, equality, and recognition (Leshem and Halperin, 2020).

While psychedelic experiences are often expressed in terms of unity and harmony, history suggests that there is a revolutionary potential in psychedelic molecules that can promote resistance to hegemony and disruption of the *status quo* (La Barre, 1938/1975; Lee and Shlain, 1985; Taussig, 1987; Musalem Nazar, 2016; Hartogsohn, 2020). The association with resistance stems from the fact that not only are psychedelics used by countercultures and colonized people but they also stimulate radical revelatory events. Indeed, after Ruqaiya had her political revelation, she attempted to disrupt the oneness of the ritual by exposing the truth about how Palestinian suffering is caused by Israelis. Since prophetic revelations have a long history of igniting political movements (Mooney, 1896/1991; Wallace, 1956; Taves, 2016), we contend that the potential of revelation-to-revolution is part of the sociopsychopharmacological repertoire of these substances.

In this sense the revelatory experience stands in a dialectical phenomenological tension with the mystical union, as they serve opposing political processes. Unlike the abstract mystical union, the prophetic revelation comes with a concrete ethical-political message. While the archetypical mystic aims to dissociate from worldly matters and transcend good and evil, it is the archetypal prophet who cries out against atrocities and injustice (Thorner, 1965). The former usually maintains the harmony and stability of the *status quo*, whereas the latter seeks to destabilize and dispute the order of things so as to achieve a greater good (Heiler, 1919; Thorner, 1965; Scholem, 1971/2011; Williams, 1983; Idel, 2000).

Drawing on Alan Badiou’s theory of events (Badiou, 2007, 2019), we analyze how participants act upon revelatory experiences during, and after, ayahuasca rituals. In his work Badiou argues that every structured situation can be understood as a *status quo*. There will, however, always be ways to engage with experiences that are part of the situation but that are excluded from the structure. Badiou defines such unusual ontological experiences as “events” that may create a rupture in the structure when recognized. Subjects, who go through this moment of recognition, face a “universal truth” beyond the structure, to which they maintain fidelity. According to Badiou, such subjects develop “fidelity” to a mission related to that truth, which they soon seek to fulfill. In this mission, they aim to include what was previously excluded from the structure. Therefore, Badiou’s events are not only revelatory but also revolutionary, as they inherently oppose any *status quo* and aim to force radical change.

In this paper, we propose that the structure of the observed ayahuasca rituals mirrors the sociopolitical structures of the Israeli state and society, which exclude Palestinians, their history, and their collective rights. We argue that the New Age emphasis on mystical union, harmony, and personal transformation in these rituals secures Israeli hegemony over Palestinians by suppressing conflict. Furthermore, we maintain that changes to the *status quo* of the ritual can occasionally occur through the resulting process of fidelity, in which the subject who had the political revelation develops a sense of mission to challenge, and potentially alter, the structure of the rituals according to the newly perceived universal truth. In this sense, such revelatory events are part of the phenomenological repertoire of psychedelics that can make them counterhegemonic by inciting resistance to the *status quo* through the subjective sense of fidelity to the event. While such psychedelically-induced fidelity has an egalitarian trajectory, it can be suppressed by agents who maintain the structure in the name of unity and harmony. Finally, as we demonstrate below, these revelatory events may lead to the expansion and diversification of psychedelic practices.

In what follows, we begin with an elaboration on Badiou’s “event,” and continue with a background discussion on the distinction between the psychedelic experiences of mystical union and revelation. We then offer a contextualization of the observed ayahuasca rituals in Israel, in which we suggest that they are structured around a depoliticized New Age culture that avoids acknowledgment of the injustice inflicted upon the Palestinian collective by Israelis, especially that which comes about through the denationalization of Palestinian citizens of Israel. Following this, we present three cases in which participants had agonizing political visions related to the excluded Palestinian existence. Attending to interventions in the rituals’ structure, we discuss the tensions that exist between the subject’s fidelity to the event and conformation to the existing structure. We conclude by suggesting that such events are part of the sociopsychopharmacology of psychedelics and that their analysis in relation to the structure can reveal different political dynamics that are part of psychedelic cultures and the psychedelic movement.

## Badiou’s Event: Revelation, Fidelity, and Universality

In *Being and Event* (2007), the cornerstone of his work, Badiou uses set theory to propose a politicized philosophy that describes the process of revolutionary events. Importantly, while such an event is a moment of recognition and revelation of a truth, its aftermath is the active procedure that leads to change. For change to take place, the event must unfold through an individual or a collective, and fidelity to the realized universal truth must follow.

For Badiou, reality is infinite, multiple, and inconsistent, without any inherent unity. Any “situation” consists of a set of elements grouped together, giving it structure. A situation can be made of anything, regardless of its modality. It can be objects, people, language, circumstances, dreams, art, concepts, and so on. The inclusion of different elements in the structured situation is an “operation” which organizes the situation into sets of elements. This operation is termed “count-as-one,” suggesting that the structure is the appearance of unity which is imposed on the situation. Some elements of the situation are included in the structure, while others are excluded–relegated to the “void.”

Usually, we experience the world only through structured situations, and the void, according to Badiou, can be thought of as the “unconscious of the situation” (2007, 66). The *status quo*, which consists of such things as ideology, language, discourse, and social norms, prevents the revelation of the excluded elements. These governing principles of the status-quo (termed also the “state-of-the-situation,” or the “metastructure”) secure the situation and keeps it from being disrupted by what exists in the void. Yet, a truth from the void can occasionally break through. Such truth exceeds the knowledge and language of the situation, and is inherently related to the void–to what is not included in the structured situation. An event is a moment that reveals what the *status quo* tries to conceal–the momentarily irruption of truth. “Event sites,” located at “the edge of the void” are where the rupture of the structure—through the event—might occur (2007, 175), and where a truth might enter. The event that momentarily amplifies the site will eventually require a restructuring in order to include the new truth. In such a restructuring, parts of the structure and parts from the void become connected to the event, and this process of establishing new connections occurs through “truth procedures.” Such procedures are revolutionary processes, as they oppose the *status quo* in an attempt to restructure the situation, and they may occur in love, art, science, or politics (2007, 335).

In the context of psychedelics, Saldanha (2007b) argues that Albert Hofmann’s discovery of LSD in 1943 can be considered a Badiouan event. In a moment of strong rupture in the understanding of the sociochemical structure of that time, the discovery of LSD resulted in a few truth procedures that transformed Western society. These procedures were attempts to include an altered state of consciousness in a structure that excluded them. The mind-liberation movement of the 1960s had a counterhegemonic universal trajectory, and the truth procedures opposed the *status quo* of that time, which was epitomized by the conformity and uniformity of the 1950s. However, we use Badiou’s theory differently. We suggest that the sociopsychopharmacological action of psychedelics can increase the possibility of events occurring. In other words, it is not just truth procedures which stem from Hofmann’s 1943 event, in attempt of including the excluded psychedelic experience, but also truth procedures which follow specific revelatory events that are recognized in specific situations under the influence of psychedelics.

Truth procedures materialize through the *subject*, who now attempts to change the situation and its structure. The subject is the “bearer” of the truth procedure and is “taken up” in fidelity to the event (2007, 406). The event prompts the subject to question the *status quo* and the knowledge instilled in the structured situation. The subject, in fidelity, is characterized by a sense of mission, confidence, and loyalty to the universal truth of the event, which leads to resistance to hegemonic structures. In this sense, one does not contemplate truth; rather, one acts, as a subject, on it. Acting in fidelity to the event, the subject does not reject the whole situation, but attempts to restructure it by connecting all of the elements that are related to the event. According to Badiou, such subjects develop an emotional attachment to their new mission. A political subject is filled with enthusiasm for a new maxim of equality, whereas an artistic subject is filled with pleasure regarding a new perceptual intensity; a loving subject experiences happiness because of a new existential intensity; and a scientific subject is filled with the joy of new enlightenment (Badiou, 2019, 76).

While truth procedures are related to a particular situation, Badiou argues that they also reveal something universal. This claim is in opposition to his postmodern and post-structuralist contemporaries. While similar to them, he embraces the emphasis on plurality and multiplicity, he also discusses the possibility of universal truths. Such conceptual and philosophical disagreements about universalism can be considered perennial (Balibar, 2020). One of the main lines of argument against universalistic discourse is that while it insists on total inclusion, it violently silences voices that claim particular marginalized identities (Taylor, 1997). In this sense, universality will always be situational and defined by a particular hegemony. Saldanha’s (2007a) description of the exclusively white trance music scene in Goa, India, is a relevant psychedelic case of this conundrum. In his work Saldanha shows how unity is achieved by preserving racial and cultural boundaries. It prevents Indians, as Others, from participating and interfering in the ecstatic oneness on the dance floor. If unity, as similarity, is prioritized over universality, then only an exclusive illusion of universality will take place.

To aspire to broader universalism, the unity of the structure must be challenged. Indeed, for Badiou, universalism is the guiding star of the truth procedure. It points the way to what is not represented by the excluding *status quo*. Badiou’s universality is always dependent on the situational particularities that it has to overcome. Therefore the subject’s fidelity to the event depends on the exclusivity of the structure, so that the subject is somewhat divided between the event and the structure (Robbins, 2010). The truth procedure is either concluded, once the existing structure is reformed, or reversed, once the structure successfully contains the event without changing.

## Ayahuasca and Revelatory Events

No one can claim with certainty when and where ayahuasca practices originated, yet it is known that most of its diffusion across the Amazon Basin occurred during the last 300 years. This was influenced by Christian missions (Gow, 1994), the rubber boom of a century ago (Brabec de Mori, 2011), and the recent expansion of shamanic tourism (Winkelman, 2005; Fotiou, 2010), neoshamanic practices (Scuro and Rodd, 2015), and the Brazilian syncretic ayahuasca churches (Labate and MacRae, 2016). Therefore, many of the characteristics of what is known today as “traditional” Amazonian shamanism were actually developed in response to colonial atrocities (Taussig, 1987) and interaction with the West (Langdon, 2013). Therefore, some of the indigenous and mestizo practices of ayahuasca are political (Taussig, 1987)^2^. These include strengthening tribal coalitions and relations to the land, as well as resistance to white colonizers (Langdon, 2016; Musalem Nazar, 2016).^3^ For example, the Siona communities have used ayahuasca as a strategy for ethnic revitalization and re-indigenization of mestizo people; ayahuasca has helped draw those who had been urbanized and acculturated back into the tribe. It also helped in building alliances with academics, political activists, and artists. Such political use of ayahuasca eventually guaranteed their ethnic survival (Langdon, 2016). In Western countercultures, psychedelics have also been historically related to antihegemonic revolutionary tendencies (Lee and Shlain, 1985). Such tendencies occurred as a result of fidelity to the LSD event, in an attempt to popularize psychedelics (Saldanha, 2007b) and/or—as we suggest here—through critical insights and revelations reached while under the influence of psychedelics (La Barre, 1938/1975; Winkelman, 2010; Luna, 2011; Labate and Cavnar, 2014; Strassman, 2014; Davis et al., 2020, 2021). Regardless of the way in which they were induced, similar revelations are known to come with the enthusiasm to deliver the message to others, which at times developed into social movements (Mooney, 1896/1991; Wallace, 1956; Lanternari, 1963; Whitehouse, 2004; Taves, 2016).

In the context of psychedelics, two notable revelatory events had a wide impact. The first is the ayahuasca-induced revelation of the “Queen of the Forest” (the Virgin Mary) to Raimundo Irineu Serra, a Black rubber tapper, who later became the founding father of the Santo Daime church (Moreira and MacRae, 2011; Orgad, 2012; Labate and MacRae, 2016). This moment marks an expansion of the practice from indigenous people in the Amazon Basin to a syncretic inclusive practice that eventually had a global impact. The second revelatory event is that of Alan Ginsberg, guided by Timothy Leary.^4^ During a *Psilocybe* session, Ginsberg had a messianic revelation of world peace, during which he tried to call world leaders on the phone while running naked through the apartment—like Archimedes in his eureka moment. In fidelity to this vision, Leary and Ginsberg decided to promote “psychedelics to the people,” beyond academic, clinical, military, and governmental circles (Leary, 1968/1995; Conners, 2010; Hartogsohn, 2020).^5^

There are important ethical considerations related to psychedelic-induced revelations and how one should follow them, as their content may be imposed on participants by facilitators (Timmermann et al., 2020). Therefore a “gentle touch” is required when dealing with such sensitive states of mind. Nevertheless, not every psychedelic insight is an *event* in the Badiouan sense. We claim that some psychedelic revelatory events are unique, as they expose something that is ignored by the facilitators or the culture in which the experience takes place. Such events can be recognized by some distinctive characteristics. They begin with a degree of separation, at times agonizing, from an exclusively structured “unity” of the group. Revealing what is excluded from the structure, these revelations are by definition counterhegemonic, since they incite resistance to the group’s *status quo*. Soon after, the receiving subject is aligned in fidelity to the event and embarks on an emancipatory mission, in which s/he seeks to spread the attained universal truth. Finally, these revelations may create a split in the structure, to achieve a more inclusive unity.

## Palestinian Identity in the Israeli Ayahuasca Ritual Structure

Ayahuasca rituals spread to Israel two decades ago through the Brazilian Santo Daime church and neoshamanic practices. Palestinians, especially citizens of Israel, joined these rituals around 10 years ago. This came along with the rise in consumption of psychedelic substances, especially in the Palestinian rave scene (Karkabi, 2021). However, their access to ayahuasca practices has been mediated through Jewish Israeli circles. While many Israelis travel to South America on post-military-service journeys (Noy and Cohen, 2005), during which they become acquainted with globalized ayahuasca networks, Palestinians have been more restricted in their international movement. This is so owing mainly to their relatively lower socioeconomic status and travel restrictions imposed on Palestinians from the West Bank and Gaza. Palestinians who joined the Israeli rituals were a small minority, as well as less experienced. Therefore, they often had to rely for support on Jewish Israeli group organizers, facilitators, musicians, helpers, and participants. In addition, some of the sites where the rituals took place were at homes and on land confiscated from Palestinians by the Israeli state and redistributed to Jewish Israelis. As the mother tongue of Jewish Israeli participants, Hebrew was the main language spoken during these rituals, yet for Palestinians it was a second language, after Arabic, or a third, after English.

Palestinians, especially novice participants, were clearly outsiders at these gatherings. They had less subcultural capital, which relied heavily on Israeli New Age spiritual jargon and social norms. More important, they were alienated from the influence of Jewish religious and Israeli national elements. In some groups such influences included a kiddush (religious Jewish blessing) before drinking the ‘‘medicine’’ or an *icaro* (Peruvian shamanic song) based on Hebrew lyrics of the Israeli national anthem, with some New Age adaptations.^6^ Furthermore, well-known Israeli songs, such as “A Walk to Caesarea,” also known as “Eli, Eli” (My God, my God), and “Mi ha’ish” (Who is the man) suggested universal and humanistic messages, but were associated for Israelis with national memorial days, such as Yom ha-Zikaron (Memorial Day for the Fallen Soldiers) and Holocaust Day. Among Israeli Jews, these songs are endowed with national and spiritual solidarity, unity, belonging, and a sense of homecoming. This “homecoming” of Israeli Jews also implies the displacement of Palestinians.

However, since the Israeli New Age culture holds an allegedly apolitical ideology (Simchai, 2009), most Israeli participants in the observed rituals positioned themselves “beyond left and right.” Claiming that “peace starts from within,” they welcomed Palestinians, based on humanistic ideology. Under this ideology, the ongoing history of colonial oppression is denied, so that the conflict is considered an “illusion,” and “the political game” should be avoided so as not to raise divisions. While downplaying sociopolitical hierarchies, inequalities, and power relations, some of these groups proudly celebrated the different cultural (Jewish and Arab) and religious (Jewish, Christian, and Muslim) background of each participant. They argued that listening to the music of other cultures “opens the heart” so that “peace is felt in the air.”

The denial of national identity of the Palestinian minority in Israel is not exclusive to the context of the Israeli ayahuasca rituals, as it matches wider political tendencies among the majority of Jewish Israelis and reflects many state policies. Defeated and dispossessed of their lands, the Palestinians who remained in Israel after the Nakba (catastrophe) in 1948 faced military rule until 1966 and eventually were granted Israeli citizenship (Robinson, 2013). Since then, however, they have been subject to Israeli policies that have denationalized, racialized, and fragmented them into religious subminorities. Thus, they became known as “Israeli Arabs” or Muslim, Christian, and Druze minorities (Kanaaneh, 2008). As non-Jews, however, they were not fully part of the Israeli nationality and hence could not become equal citizens in Israel, as long as Israel remains defined as the nation-state of the Jewish people. Although this limited assimilation of the “Israeli Arabs” retained Palestinian national identification among some, it led others to adopt an integrative politics of civil equality, in the hope of individual acceptance into Israeli society (Ghanim, 2020). While national exclusion and racism toward Palestinians has only increased in the last decade, backed by a series of undemocratic laws issued in the Knesset (Adalah, 2017), economic liberalization in Israel increased employment opportunities to a growing middle class of Palestinian citizens (Sa’ar, 2016).

It is within this context that we understand the appeal that Israeli ayahuasca rituals have for middle-class Palestinian citizens of Israel. Along with moments of intercultural and interfaith unity, the rituals promised New Age universalist spirituality, based on the allure of a collective experience of “oneness” and “identity dissolution” (Roseman et al., 2021). For example, Sawsan, a Palestinian citizen of Israel, described what drew her to these rituals: “We are all together, no Jewish, Arab, Christian, Muslim. Everything fades. All that nonsense is laid low and all that is left is acceptance and love.” With the same attitude, many other participants, Israelis and Palestinians, asserted that ayahuasca strips away identities and that everyone can connect “on the level of the soul,” as “human to human.” However, relating to difference, Sawsan explained that each language and culture has its own “frequency” and that the union of all frequencies is “the white light.” Here, diversity comes to express oneness, a feeling of connection and harmony. A sense of tribal connection and love develops among group members, but it does not guarantee equality or recognition, mainly because the looming political tension is kept strictly silent.

As research on the New Age culture in Israel suggests, the problem is that “blindness toward ethno-national identity reinforces identification with a self-evident hegemonic perception, thereby leading to the exclusion of peripheral groups such as indigenous populations” (Simchai and Keshet, 2016). While it is easy to unify Jews and Arabs as offspring of the same ancestral patriarch who worship the same God, it is much harder to unify Israelis and Palestinians by their attachment to the same land. Therefore, diversity is included in the ritual as long as it is apolitical and non-conflictual. Similarly to some dialog groups between Palestinians and Israelis that do not involve the use of psychedelics (Maoz et al., 2002; Abu-Nimer et al., 2007; Maoz, 2011), these rituals avoided dealing with the violent atrocities that Israelis have inflicted upon Palestinians. The displacement, dispossession, and colonization of Palestinians, both past and present, was silenced and ignored. The “irony in harmony” is that such pseudo-equality can create false perceptions that eventually inhibit actions that might lead to actual equality (Saguy et al., 2009; Hasan-Aslih et al., 2019). The focus on harmony in the observed groups led to the preservation of ethnonational power relations in these rituals, just as they are in the wider social and political structure in Palestine/Israel. Driven by a settler-colonial exclusivist ideology, which is justified by theological righteousness, most Jewish Israelis are reluctant to give up privileges and share equal civic and political rights with Palestinians (Wolfe, 2016). Thus, the experience of “oneness” challenges neither the Israeli political structure nor the Israeli structure of ayahuasca rituals; rather, it stabilizes it at a *status quo* in which Israelis dominate Palestinians.

In Badiou’s terms, Palestinian identity is kept “on the edge of the void” (2007, 176) to preserve harmony. It is a potential event site that is not connected to other parts of the structure. In this sense, Palestinian language, music, and religions can be part of the ritual structure as long as they are not explicitly considered Palestinian. For example, Arabic music can be part of the structure through New Age multicultural universalism. Similarly, Muslim prayers can be included through perennialism, which argues that the mystical union is at the core of all religions (Huxley, 1945). Yet, what is completely excluded from the structure is the recognition of Palestinian identity as the locus of the ongoing Israeli history of injustice. It is this history that makes the event site unstable, occasionally allowing the experience of radical revelatory events.

In the following sections, we detail three cases in which revelatory events were related to the collective trauma of the Palestinian people. Induced by the consumption of ayahuasca, these were moments in these rituals in which the stabilizing oneness was ruptured. We demonstrate how the affected subjects were first separated from the group and then driven by agony and anger that ignited their truth procedures. Taken up in fidelity to the visionary event, they each attempted to intervene in the rituals’ structure by delivering an emancipatory message, in the form of a song, to the rest of the group. While their message came as a demand to recognize the injustice that Jewish Israelis have been causing Palestinians, it also aimed to achieve inclusionary universalism, in the rituals and beyond. Although at times these subjects were somewhat successful in changing the rituals’ structure, they often had to act outside of it to establish a more accepting ritualistic structure for Palestinians.

## Materials and Methods

The three case studies presented in this manuscript are part of a larger research project that examines relations between Palestinians and Israelis in the field of ayahuasca rituals. For detailed methodology section see Roseman et al. (2021) and its supplementary section. In short, most of the data for this project was gathered in 2018–2019 through 31 in-depth interviews: 13 with Palestinians (five women, and eight men; nine have Israeli citizenship and four live under occupation in the West Bank) and 18 with Jewish Israelis (eight women, nine men, and one non-binary person), all between 28 and 59 years old, who participated to various extents in these mixed rituals. Interviewees belonged to five different ayahuasca groups, mostly facilitate by Israeli Jews. Facilitators and musicians lead the ritual with music, yet most rituals had participatory elements as well. There were moments in which the group sang together, and by the second half of the ritual participants were able to share their own music or prayers. Rituals included around 20 participants in average. The first author had an initial connection to some interviewees, which then introduced him to others through a snowball manner. However, the interviewees sample was a *purposeful*, so that we attempted to choose diverse informants in order to achieve reliable and generalizable data (Mason, 1996).

In-depth semi-structured interviews were conducted in Hebrew, Arabic, and English^7^. Each interview lasted from one to two and a half hours. All interviews were recorded and transcribed. The microphenomenological interview technique was used whenever an interviewee reported important experiences and events from the rituals that had political implications (Petitmengin, 2006). This technique was crucial for “zooming-in” on small details of the revelatory events presented in this paper. Author 1 also conducted participatory observations in five rituals of different such groups for complementary ethnographic data collection. Furthermore, follow-up interviews were conducted 1 year, and 2 years, after the first interview for the three interviewees described in the case studies.

The analysis was based on the grounded theory approach (Glaser et al., 1968). This approach emphasizes hypothesis-free bottom-up generation of concepts and themes. In line with this approach, several stages of analysis were undertaken (Berg, 1988/2004; Strauss and Corbin, 1990; Shkedi, 2003, 2019). The first phase included a thematic analysis of the interviews, which revealed thematic categories. Through a process of reading and re-reading the interviews, the number of categories was reduced by combining similar categories and focusing on those that emerged as most relevant. These categories were scrutinized again for centrality (repeated appearances across interviews and observations), for the connections between them, and for their relevance to the study and the questions it addresses. The software Narralizer was used to organize and codify the interviews (Shkedi and Shkedi, 2005). Narralizer is a simple software for organizing and structuring qualitative data, and no automatic analysis was conducted.

In a previous paper from the same dataset, fifteen conflict-related revelations were identified (Roseman et al., 2021). Out of which five were defined as political revelatory events based on the theoretical framework of the current paper. The three revelatory events that are presented here were chosen based on having a detailed description of both the event itself and the developments that followed it. The description of the case studies is mainly based on the three interviewees, yet some information is used from other interviewees who attended the rituals in which the revelatory events took place. The ethnographic analysis of the context section above is based on all of the gathered data.

## Regaining a Self-Denied Self

Identifying as an “Israeli Arab,” Khalil grew up in the Galilee and went to a Jewish Israeli school, where he was denationalized and assimilated to become very critical toward Palestinians. Looking back, he says that he “was a person who denied his own Arabness.” A supporter of right-wing Israeli politics, in his youth, he even wanted to serve in the Israeli army. Dispossessed of his national history and identity, Khalil reached ayahuasca rituals when he was in his 50s. He wanted to heal a personal trauma inflicted upon him by a childhood friend and heard that ayahuasca can help people purge their trauma. As the healing had already taken place in his first session, he became a regular member of one of the Israeli groups that organized ayahuasca rituals.

After several sessions with that group, Khalil was invited to a ritual in a private house located in a Jewish Israeli town. As was evident from the architecture, the house had formerly been owned by a Palestinian family who, like many other Palestinians, had been displaced during the Nakba. The current Jewish owners enjoyed hosting many rituals in the large and accommodating house. Guided by the Jewish Israeli shaman that Friday evening, this ritual was somewhat different, as it had a major Jewish influence involving the welcoming of Shabbat. Jewish participants were all dressed in white and were singing Jewish religious songs. Khalil came in jeans and a colorful shirt, like the other three Palestinian participants in the ritual, and was asked by the organizers to change his clothes. This contrast of colors intensified the distinction between the Palestinian minority and Jewish majority of participants, which is probably what summoned Khalil’s confrontation with his self-denied Arabness from “the void.”

While the group was singing the traditional Shabbat song “Shalom Aleichem” (Peace be on you, in Hebrew), Khalil decided to go out to the porch. Separated from the group, he sat under a vine tree that reminded him of his grandfather’s house. An intense light started flashing. Sitting outside alone, fear took over him. Khalil felt as if he was losing his mind and would never return to his usual self. He imagined himself becoming a “junkie in the streets,” and was wondering “what will I tell my wife and how will I look my children in the eye.” His fear intensified his sense of alienation and separation from the world, believing “that no power can release me from where I found myself.” Such separation is a necessary step for an Badiouan intervention to take place (Badiou, 2007, 201, 209). While lifting his head to the sky to pray for God’s help, Khalil had a vision of an old Palestinian couple in traditional Arab clothing, sitting in front of him on the same balcony and drinking coffee. They were the previous Palestinian owners of the house where the ritual was taking place. The woman told him: “I know you went to an Israeli school—you hate everything to do with Arabs, and you have anger. But the story is different. You heard only one side, but you haven’t heard our side. You are in our house now and we are not here. We were evicted.”

Khalil deeply identified with the old couple’s pain. The revelation confronted him with collective Palestinian traumatic memories of the ongoing effects of the Nakba, which he had previously avoided. Angry at himself, Khalil felt deceived by the Jewish Israeli narrative. Loyal to his vision, he returned to the ritual with the aim of disrupting it. In anger he sang an old Arabic Andalusian song, “Lamma bada yatathanna” (When it began to unfold), in order to declare that this was “in fact a Palestinian house… whether we like it or not.” The psychedelic event led to his recognition of the injustice that Israelis perpetrate against Palestinians, after which, in Badiou’s terms, he felt compelled to intervene in the “structure” of the ritual and modify it. Singing in Arabic was a political act for Khalil, but the reaction of those responsible for maintaining the structure of the ritual was swift. When Khalil raised his voice, the shaman invited him to sit next to him and played the joyful song “Bint al-shalabiya” (The pretty girl) by Fayrouz, a famous female Arab singer from Lebanon. Khalil recounts the excitement: “I am mad about her [Fayrouz]. Suddenly, the shaman starts singing in Arabic. We were thirty people and everybody got up to dance and hug…. I was shocked; hugging and dancing, Jews and Arabs! It was wonderful, wonderful…. That’s where the journey started.”

Khalil’s fidelity to his vision, as we understand it, was expressed through an angry musical interruption. However, this was not enough to change the structure of the ritual that evening. Although it seems that the Jewish-oriented ritual suddenly became Arab, as people cheerfully danced to Fayrouz’s song, it became clear to us that the shaman successfully diverted attention from the national political grounds expressed in Khalil’s anger to culturally safe grounds. The shaman prevented Khalil’s truth from disrupting the harmony of the ritual with Palestinian memories of displacement from the Nakba.

However, Khalil considered his bringing himself to sing in Arabic at the Israeli ritual an outstanding achievement, a pivotal moment when his “journey began.” “Born again” in this event, he felt his brain was “clean to receive the truth.” Attuned to the universal truth that he had attained at the event, Khalil tattooed his chest with a sentence in praise of the land, taken from a poem by Mahmoud Darwish, the Palestinian national poet. This helped him reassemble elements of his Palestinian identity, as he confessed: “I used to be very radical right wing in my opinions…. [But] I have made peace with the Arab in me, with being a Palestinian Arab.”

Khalil remained loyal to the event after the ritual was over. Uncomfortable with joining Israeli rituals in which he was part of a minority, his fidelity was directed toward the expansion of ayahuasca to Palestinians. He began organizing rituals for Palestinians only, to which he introduced Arab musical instruments, such as the oud and qanun. As a result he became a key figure in the diffusion of ayahuasca to Palestinians living in Israel. Still, he continues to hold a strong universalistic worldview, arguing that what he learned from his event is not specific to Palestinians. This conundrum can be reconciled through Badiou’s conceptualization of universalism, in which existing structures are restructured to include the excluded particulars. Khalil’s mission to expand ayahuasca to Palestinians led to an empowering and intimate circle, in which they are not dependent on Israeli mediation. Although open to Jewish Israelis, Palestinian ayahuasca rituals offer an alternative structure in which they can reclaim the Arabic language and culture as part of their Palestinian heritage, and not as a denationalized discourse of multiculturalism and interfaith dialog. Such an alternative structure of ayahuasca rituals, as we demonstrate in the following section, also allows Palestinians to produce a safe space with a new cultural vocabulary, where they can share the pain and anger that they experience as a result of the Israeli settler colonial structure.

## Diversification and Politicization

Working at a regular nine-to-five job in finance, Ruqaiya kept away from politics. Although growing up in an Arab family in a village in the north of Israel, she felt an outsider in her Palestinian culture. She married young, in an arranged marriage to an older man, but fell in love outside the marriage. This led to traumatic and violent events. Divorced with two children, Ruqaiya first participated in ayahuasca rituals when she was in her 40s, or as she explains: “Ayahuasca reached me. She searched for me.” During that period, Ruqaiya was at a low point in her life. She was “a person who saw everything in black, with no point in life.” Ayahuasca, her “teacher,” showed her that her trauma was “a gift.” It taught her about “the depth of life,” and that she was able to “adapt to any place and any situation.” Ruqaiya soon became a therapist, “a wounded healer,” as she describes it, focusing on treating trauma.

Four years into her participation in Israeli ayahuasca rituals, she had her major life-changing revelation. As recounted at the opening of this article, it was on the Jewish holiday of Yom Kippur (Day of Atonement). She joined her regular group, her “tribe,” yet was the only Palestinian in the ritual that night. During the ritual someone sang a song about Maria. She felt as if “Maria entered” her, and she soon began vomiting. She then received many insights about Amal, her elder daughter. Similarly to Khalil, Amal attended a Jewish Israeli school. Coming of age at the time of the event, she was considering joining the Israeli army. Although a Muslim, Amal also fasted on Yom Kippur that day, at the house of her Jewish friend. Strongly assimilated into Jewish Israeli society; her Palestinian identity was, to use Badiou’s phrasing, on “the edge of the void.” In her vision, Ruqaiya saw Amal with some darkness closing in on her, “disappearing into an abyss.” She had to reach out to Amal and pull her out of that darkness and away from her confusion. At that point, as Ruqaiya recounted, “something closed and I was higher. *Al-Fatiha* [the opening verses of the Quran] came out, and another strong frequency was opened.” This vision separated her from the rest of the group and allowed the intervention to take place.

While singing *al-Fatiha* to the rest of the group, Ruqaiya had a strong revelation in which she saw Palestinian and Israeli mothers sacrifice their own children to war, while ‘‘*Pachamama*’’ (Mother Earth, in Quechua; a term popularized in neoshamanic practices) absorbed the bloodshed. Ruqaiya experienced the pain of the land.^8^ The historical vision continued from past traumas into an apocalyptic warning from a potentially disastrous future. As she explained, the vision of mothers who sacrifice their children was related also to her daughter’s intention to join the Israeli army.

Ruqaiya’s singing and vision were simultaneous. She was singing out of agony about the vision, as she recalled: ‘‘The voice came from the center. I cannot say it was in the voice of God, though it was the voice of a messenger.’’ When she sang, she ‘‘released a frequency of anger,’’ and it felt as if she were telling the other participants to ‘‘listen and awaken. There is a battle here between light and dark. The dark is growing stronger than the light, so understand where we are now.’’ When asked about the meaning of *al-Fatiha*, Ruqaiya chose a universalistic interpretation. She said that it is about not getting lost: ‘‘All those who go by certain laws, which are illusory and fake, separate from the human itself, from humanity, from what we are. We are human.’’ After *al-Fatiha*, she sang, in Hebrew, a song that was popular among the group members, but she added a verse in Arabic.^9^

While she was singing, there was an intense feeling in the room, and many participants were vomiting. In relation to the group, Ruqaiya said: “I felt each one, each and every one [in the group]. Where they were with their fear, doubt, and ego. The [protecting] net that each person places himself in. The defense of the illusion.” Her singing was a pivotal moment in the ritual for other group members, but only few understood the message, as she explained: “Many friends deny it [the message]. They don’t want to deal with it…. So who is doing reparation [on Yom Kippur]? No one. They [Jewish Israelis] are just fasting.” Ruqaiya’s fidelity to her vision was counterhegemonic. As a result, she delivered anger in her song, which we believe was related to a political truth that was concealed by the structure of the ritual. Ruqaiya’s critical insight regarding her own ayahuasca “tribe” was that it replicates the power dynamics that exist in larger reality. However, as in the case of Khalil, her attempts to intervene resulted in denial by the representatives of the ritual’s structure.

While the prophetic interventions of Palestinians channel political anger during rituals, we noticed that New Age spirituality often comes to support the Israeli structure of political denial. For example, Nir, an Israeli shaman, suggested that if political revelations take place during the ritual, they are part of the “shadow work” that one needs to go through. In his instructions, Nir meant that once the political “shadow” becomes conscious, a person will be less guided by it. In this sense, a political revelation is personal, meant only to reveal restrictive identities and traumas to oneself, and through this awareness one can work toward liberating oneself from such restrictions. This argument could be used to reverse Palestinian revelatory truths, as it suggests that their expression of anger was needed for their cathartic personal healing, but not as a message to the group. This reversal is supported by New Age ideology in which “the self” is at the center of transformation.

Nevertheless, the revelation that Ruqaiya had during this ritual developed into a sense of mission and meaning in her life. However, we observed that the intensity of her fidelity to the event was reduced, in comparison to the initial intervention, and remained mostly related to the setting of the ayahuasca practice. She explained: “I feel I am an ambassador of the Arabs in that I started to say, ‘Yes! We are here. We have a voice and opinion. We have life. We are allowed to live. Not only if the Jews allow it.”’ Ruqaiya took her destiny in her own hands and began singing to liberate the Arabic language from the oppression of the Israeli state and patriarchy. According to her, it is “the woman who brings revolution and change.” This realization came in fidelity to her vision, yet her self-inquiry is still in process: “How to do this? I am checking, receiving answers, in order to extricate the woman who is the educator of all ages and the one who brings children into the world.” Ruqaiya’s transformation was reflected in Amal, her daughter, who eventually decided not to join the Israeli army, conceivably owing to the event. Considering her singing an empowering political act, Ruqaiya encouraged other Palestinian women to join her rituals and “find their voice.” Similarly to Khalil’s fidelity, she sought to expand and diversify the ayahuasca rituals, to make them more egalitarian and inclusive for Palestinians.

Her politicization did not, however, happen at once. Worried that people would not understand her, it seems that she was divided between fidelity to the event and belonging to the structure. As time passed, and as she remained in fidelity to her vision, the structure around her changed, and she was able to admit to herself that her revelation was political. Throughout her process of political self-exploration, she met like-minded people who could understand her mission. Furthermore, two Jewish Israelis who participated in the Yom Kippur ritual received the message of her song and became musical companions in the rituals that she facilitated.^10^ Gathering a small group of people around her, Ruqaiya has not only kept her fidelity alive but has been seeking further connections to form a larger social movement. In this sense, she has been trying to establish a politicized collective that she hopes will have a larger impact.

On a few occasions since her event, Ruqaiya has attempted to deliver explicit political messages during rituals. For example, during a magic mushroom ritual, which Ruqaiya co-facilitated with a small group of spiritual activists, Adi, a Jewish Israeli woman, announced to the group that she has just decided not to move to Portugal. Addressing both Israelis and Palestinians, she declared: “This is my home and these are my people.” Soon after, in a moment of inspiration, Ruqaiya sat in the center of the circle and spoke in archaic Hebrew, which resembled a biblical text. Accusing the “children of Israel” of losing their way, she stated that “those who were liberated by Moses in the past, have now became Pharaoh [to the Palestinians].” Such direct verbal phrasing of the duality of the oppressor/oppressed is a radical break from the previous structure, which sanctified oneness. As part of the inquiry into her truth, we believe that Ruqaiya has been replicating her event and her initial intervention in other contexts. In doing so, she reconnects with the excluded Palestinian identity, by using other parts of which are in either the Israeli structure (Arabic language and music, Islamic religion) or the void (right to the land, historical injustice). Hence, according to Badiou, she became a “subject” through which the restructuring occurs. She admits though that this is not a simple task and that the integration of her event is challenging as it requires replacing the “old language” with a “new language” that fits the event. Such tension is inherent to the truth procedure, as the restructuring cannot be dependent only on the language of the previous structure.

Ruqaiya’s politicized prophetic deliverance in rituals suggests that she is seeking the reformation of New Age culture so as to make it more politically engaged. While borrowing and decontextualizing different religious practices is central to New Age spirituality, Ruqaiya reconstructs ayahuasca rituals in Palestine/Israel in a political frame. As she puts it, she seeks to “wake up” those who “prefer not to see.” Her belief that “all is one” is now aligned with a mission, as her call for action is mobilized to achieve equality at large, beyond the Israeli ritual structure.

## The Divided Subject

While the event site is related to the Israeli subjugation of Palestinians, Jewish Israelis can also experience revelatory events that lead to political awakening. Today in his 30s, Amos grew up in a middle-class Jewish Israeli family with a left-wing orientation. Never politically involved himself, he ended up joining the Israeli army as a soldier in an elite unit. He decided to join a combat unit not for patriotic reasons, but “to be a man.” During his service, Amos went through a few traumatic events in the West Bank, in which his life was under threat and his comrades were severely injured. After his army service, he felt “very tense” when he heard Arabic spoken near him. After some of his classmates from high school died in combat in the Second Lebanon War (2006), he decided to leave the country to travel. Like many other Israelis, he went on a long journey to India and elsewhere to overcome the harsh memories of military service. Amos had his first experiences with psychedelic drugs as a backpacker-musician. Regularly returning to Israel for occasional jobs, he then became part of the ayahuasca milieu there.

Looking back to his first encounters with Palestinians at Israeli rituals, he confesses that he was initially judgmental toward their appearance. Not conforming with the Israeli hippy dress code, they were, according to him, too neatly dressed and showed off their high socioeconomic status. As he recounts half jokingly: “I remember a [Palestinian] guy who came to a ceremony in leather shoes, jeans, cell phone, BMW keys, pack of cigarettes, and even when he changed clothes, everyone else was in hippy clothes.”

His life-changing revelatory event took place in a session in which he came across Palestinians from the West Bank for the first time in such a setting. As the Palestinian participants began to cry during the ritual, Amos’s vision was triggered:

Suddenly, the ayahuasca showed me them [the Palestinian group] as a separate unit within us [the Israelis in the ritual]. Another one [Palestinian] began to cry. It took me automatically to the madness of the pain of a whole people…. I felt connected to that pain. I caused the pain of their people. I began to break. I couldn’t stand to hear them crying. She [ayahuasca] began showing me so much. I can’t describe it visually, just this crazy pain, and hate, and crying for the evil they experienced. It built up and up, until there was a cut.

The collective cry of the excluded group of Palestinians separated Amos from the room into his own vision, whereby he had a very detailed flashback of his army service. He saw himself making a casual house arrest of Palestinians, “one of dozens, maybe hundreds,” as he confessed. He saw himself with his unit breaking into the house, interrogating the Palestinian family, and then leading a man into a military jeep. Soon Amos had a “cut” in the revelation and he re-experienced the same incident again. This time, however, he experienced the moment from the side of the family, feeling their pain, panic, and heartbreak. Observing himself from the other side, he described himself as looking like “Robocop,” or “like someone from a film about Nazis.” When the revelation ended and Amos returned to the ritual, he felt intense anger and guilt. “That was the point in my life where I most hated myself,” he later admitted.

After recognizing the pain that Palestinians go through, Amos became devastated during the ritual. He began singing with much confidence, even though until then he had been embarrassed to sing by himself in rituals. Delivering the message through song broke the barriers of his shyness. Amos was taken up in fidelity. He requested from the facilitators permission to sing relatively early in the ritual. Thus, he intervened in its regular structure. Amos remembers that he did not sing beautifully, but that there was something authentic and honest in the moment. Amos considers the song to be a direct continuation of the revelation. He mentioned that it felt like the “room was electrified,” and “the world vibrated with me.” He felt as if he embodied the role of a “preacher” delivering a crucial message of truth to the group. “I felt it [the song] was strong, stronger than me, and it came from a black *void* in the ritual,” Amos’s words interestingly resonate here with Badiou’s terminology.

The song that Amos sang was “Mekomi kadosh” (My place is sacred), a Hebrew song influenced by Native American peyote-ritual music. The lyrics had a universal message for him, stating that everyone’s connection to the land is sacred and everyone’s voice should be heard. Amos, however, gave it also a localized political meaning, in which he expressed his relationship with Palestinians and their mutual connection to the land. Up to that point most of the songs in this specific group were in Spanish, English, and Portuguese, since the facilitator was European. Singing in Hebrew, Amos felt that the song was “stronger than me.” Although participants are usually encouraged to stay in their place during the ritual, two of the Palestinian participants came and lay down next to him. According to Amos, this was when “my journey of healing my relations with them [the Palestinians] began.” The song ended with a long silence, which allowed people to reflect.

Rashid, who went to lie down next to Amos while he was singing, also found the courage to sing later in the same ritual. It was the first time that a Palestinian sang in Arabic in this particular group. As with Khalil and Ruqaiya, the introduction of both Hebrew and Arabic to the singing indicates that the fidelity to truth led to the expansion of the ayahuasca ritualistic practice to include local identities, as well as to greater universality. Eventually, Amos befriended the Palestinian group in the ritual. He left the reserve army and began to learn Arabic. He also developed great interest in Palestinian culture and history. However, in addition to his connection to Palestinians, Amos argues that this specific ritual made him less judgmental and more compassionate toward all humans. This inductive reasoning suggests that through a particular recognition, something universal was revealed to Amos.

Another indicator of an essential restructuring of rituals toward universality is related to the connection to the land, a theme that appears in all three of the events and fidelities described in this article. In her vision Ruqaiya identified with the pain of the land; Khalil’s new tattoo expresses his belonging to the land; and the lyrics of Amos’s song were about everyone’s sacred place on the land. Reestablishing the connection of Palestinians to the land is a crucial diversion from the Israeli structure, toward both a particular acknowledgment and a more inclusive universalism. Such universal aspirations require resistance to the Israeli settler-colonial project that supports the Zionist narrative calling for the “redemption of the land” from the Palestinians who “occupy” it (Suleiman, 2002).

Although Amos was initially in full fidelity to the event, like any other subject he later became divided between his fidelity to the event and his belonging to the structure. As with any Badiouan event, the forces of the structure are always at work to reverse the event’s truth. In Amos’s case, such a reversal is exemplified by his later reinterpretation of the event based on the previous structure. In the first interview with him, he interpreted the song “Mekomi kadosh” as an expression of Palestinian and Israeli connection to the land. This interpretation is loyal to the event, as it recognizes the Palestinians’ right to the land, which is something that the structure denies. However, in a second interview with him, that took place a year later, Amos gave the lyrics another interpretation associated with self-acceptance. He explained: “There is this thing from the North American Dakota tribe about being a man. OK, so you cried, apologized, and you are also *evil*… now it is time to move forward. What does it mean? Exactly like in the song, your place is your place and you cannot even control it. You are part of a story and your part is sacred.” We argue that this new interpretation is a diversion from the truth attained during the event, and does not challenge the structure, as it frames the song in a holistic interpretation that accepts good and evil as “sacred.” Thus, the later interpretation waves off Amos’s feelings of guilt and desire for reparation. In this sense, it is also compatible with Ruqaiya’s critique of Jewish Israelis who prefer “not to see.”

Aligned with this interpretation, Amos also said that he learned from “the medicine” that friendship with Palestinians is valuable for him, as “*peace* occurs human to human,” so that he need not “worry about the macro.” While he indeed overcame some of his stigmatization and fears of Palestinians, it seems he maintained an apolitical position that is centered on harmony and friendship, without actively taking responsibility for and acting against the structure of injustice against Palestinians that he is part of. For example, he was upset every time one of his Palestinian friends from the rituals critiqued the “Zionist occupier” on social media. He felt that such outspoken criticism was not aligned with their friendship with him. Instead, such Palestinian expressions of anger seemed to him a contradiction of the harmony and unity that they experienced together in the ritual.

We must ask then, what happened to Amos’s fidelity to his event. Why was he diverted from his initial conviction in a politicized universal truth to a more comfortable position that does not challenge the Israeli structure? It is important to note here that Amos has been a helper and musician in the Israeli rituals. Hence, he is a central representative of the structure, unlike Ruqaiya and Khalil, who were marginal to the structure at the moment of their event. In this sense, we argue that the structure was stronger than the event for Amos, so that his fidelity was eventually reversed. Moreover, while Khalil’s and Ruqaiya’s fidelity was ignited by anger, Amos’s fidelity as a Jewish Israeli was ignited by guilt. As such, guilt carries a strong psychological burden, especially when not directed toward reparation. Six years after the revelatory event, Amos went to an MDMA-assisted therapy session and revisited the haunting memory that appeared to him at the event.^11^ A few weeks after the MDMA session, he said that it had helped him to overcome the memories and to continue with his life.

## Conclusion

In this article we argue that Badiou’s theory is relevant to understanding the sociopsychopharmacology of psychedelics and their political implications. Through the three cases presented above, we have demonstrated how ayahuasca can induce political revelatory events that raise matters related to the oppression of Palestinians and are suppressed by the Israeli ritual structure. These events lead the receiving subjects to develop fidelity to attained truths about the particular context of Palestine/Israel. Although particular, these truths have a universal realization, as they aim toward inclusion and recognition of elements that fall outside the hegemonic structure. The revelatory procedure does not stop at this, as the fidelity to an event’s truth compels the receiving subject to develop and then act upon a sense of mission and confidence to oppose and possibly change both the ritual’s structure and the larger sociopolitical structure. The sociopsychopharmacology of the substances leads the subjects to gather others around the egalitarian revolutionary truth procedure to support the restructuring, by changing the existing rituals or establishing alternative rituals, albeit more inclusive and universal. Through this sense of mission, the subject, in fidelity to the event, supports the diversification of the ayahuasca practice to represent the event site in the structure. However, persistence and commitment to the event’s truth are required for success, since agents of the structure constantly attempt to reverse this truth and place it back in its initial framework. We believe that such a reversal happened in one of our observations, in which Amos was too involved in the structure and was unable to emotionally sustain active fidelity to his event’s truth.

The contrast between the harmonious unity of the Israeli structure and the injustice that Israelis have caused Palestinians amplifies the event site that lies on the border between the structure and the excluded “void” and eventually makes the rupture possible. Therefore, unity and revelatory events are not necessarily independent phenomena, but are in relation to each other. This suggestion can clarify why mystical practices are considered unitive and/or liberative (Ferrer, 2002). Religious rituals with high emotional intensity can lead to a strong identity fusion among group members, but they cannot easily maintain doctrinal orthodoxy, as this mode of religiosity is experiential. Personal revelations will eventually lead to a schism within any doctrine (Whitehouse, 2004; Whitehouse et al., 2014).

The revelatory rupture with the exclusive structure leads to revolutionary motions that diversify the psychedelic practice. In other words, the movement of psychedelic practices outward in a universal manner is also dependent on counterhegemonic revolutionary motion inward toward the structure of the practice itself. Leary’s charismatic fidelity was not just to the 1943 LSD-event, but also to the fact that Ginsberg’s event and intervention liberated psychedelic practices from institutional exclusivism and Huxley’s elitism. Leary was Huxley’s protégé and a professor at Harvard, and hence was deeply embedded in the exclusive structure of psychedelic practices of his time. Ginsberg came from the “void,” ruptured that exclusivity, and ignited a universal motion by arguing that mystical mind states should be accessible to all (Conners, 2010). In a similar sense, Ruqaiya and Khalil diffused the ayahuasca practice not just because they wanted to spread altered states of healing to Palestinians, but also because counterhegemonic fidelity intervened in Israeli exclusivism. Ruqaiya and Khalil drew connections between the Palestinian event site and Arabic music and language, which are already part of the structure but not connected to Palestinian national identity. Ruqaiya has also succeeded in realizing the “frequency of anger” from her initial intervention by introducing critical political engagement into New Age spirituality.

Truth events are crucial for the emergence of counterhegemonic egalitarian sentiments in psychedelic practices. However, persistence and commitment to the event’s truth are required to achieve change, since agents of the structure constantly attempt to reverse the fidelity to this truth and place it back into its original framework. When fidelity is reversed, the revolutionary charisma is routinized. In service of the structure, New Age ideology can reverse political truths by fetishizing harmony, unity, and the self, so as to deflate collective political action. The affected subject is then torn between fidelity to the event and belonging to the structure. Amos experienced just such a reversal of fidelity. He was too involved in the structure and could not emotionally sustain the sense of active responsibility to his event’s truth. However, the attained truth does not fade away so easily, as the subject cannot forget the radical event experience. Unable to act upon their truth, such individuals might consider dropping out of the structure instead of trying to change it.^12^ Given the strength of Israeli ritual structure and sociopolitical structure, it is not surprising that many of our interlocutors, constantly consider migration abroad, to a friendly community in “nature,” frequently in Portugal, with the hope of building a new structure that is rid of political tensions.

At other times, the dynamics between structure and event can subvert the enthusiasm of the rupture in service of the structure itself. Fidelity seems to be common across many psychedelic practices, yet it can become pseudofidelity if the structure successfully claims it. Such pseudofidelity can be clearly recognized by its revolutionary tendencies, but a lack of universal aspirations. It looks like fidelity, but it aspires to restrictive unity. The influence of “conspirituality” (Ward and Voas, 2011)—defined as the overlap of conspiracy theories and new age spirituality—on psychedelic practices is an example of how counterhegemonic tendencies can be easily manipulated.

Though we have discussed only political events in this article, Badiou’s event also relates to love, science, and art. Attending to these other analytical possibilities may offer new insights into psychedelic experiences. Badiou suggests, that “the militant of a truth is not only the political militant working for the emancipation of humanity in its entirety. He or she is also the artist-creator, the scientist who opens up a new theoretical field, or the lover whose world is enchanted” (2007, xiii). To analyze such events, it is important to examine the revelatory moment in relation to both the structure in which it occurs and the fidelity that ensues.

## Data Availability Statement

The datasets presented in this article are not readily available because the data contains personal information that cannot be easily anonymized. Requests to access the datasets should be directed to LR, leor.roseman13@imperial.ac.uk.

## Ethics Statement

The studies involving human participants were reviewed and approved by Joint Research Compliance Office at Imperial College London and the Imperial College Research Ethics Committee (ICREC reference 18IC4346). The patients/participants provided their written informed consent to participate in this study. Written informed consent was obtained from the individual(s) for the publication of any potentially identifiable images or data included in this article.

## Author Contributions

LR designed the study, collected and analyzed the data, and wrote the manuscript. NK supervised the analysis and wrote the manuscript. Both authors contributed to the article and approved the submitted version.

## Conflict of Interest

The authors declare that the research was conducted in the absence of any commercial or financial relationships that could be construed as a potential conflict of interest.

## Publisher’s Note

All claims expressed in this article are solely those of the authors and do not necessarily represent those of their affiliated organizations, or those of the publisher, the editors and the reviewers. Any product that may be evaluated in this article, or claim that may be made by its manufacturer, is not guaranteed or endorsed by the publisher.

## Footnotes

1 All interlocutors’ names are anonymized, and identifying information has been slightly modified to maintain privacy.

2 Taussig (1987) argues that ayahuasca visions of the historical collective trauma of indigenous people fueled its revolutionary potential in the amazon.

3 Similar patterns were observed with peyote in Central and North America (Mooney, 1896/1991; La Barre, 1938/1975).

4 Leary was informed by Ginsberg and William Boroughs as to how the Amazonian curandero (a healer who uses plant medicines) facilitates yagé rituals. Leary then played this ritualistic role.

5 While breaking off from institutional exclusivism and Huxley’s elitism, this schism occurred in parallel in multiple places—e.g., Ken Kesey and the Merry Pranksters’ West Coast psychedelia (Wolfe, 1968/2008; Saldanha, 2007b).

6 Rewording songs is a common practice in Israeli New Age culture, see Simchai (2009).

7 Interviews in Hebrew were conducted by the first author, in Arabic by Antwan Saca, and in English by Natalie Ginsberg.

8 Three other Palestinian women who were interviewed had a similar revelation, in which the earth absorbed the blood of war. For an ecofeminist perspective on the conflict, see Eisler (1987/1998) and Roseman et al. (2021).

9 The song was “Kanfei ruah” (Spirit wings), whose lyrics were written by Rabbi Abraham Isaac Kook, one of the fathers of religious Zionism.

10 One of them also convinced his daughter not to join the Israeli army, owing to Ruqaiya’s intervention.

11 MDMA is an empathogenic drug that is used for the treatment of post-traumatic stress disorder (PTSD) in clinical trials (Mithoefer et al., 2019).

12 See “The Houseboat Summit” for a conversation between Leary, Ginsberg, Watts, and Snyder about political activism vs. dropping out (Watts et al., 1967).
